# Does flip-flop style footwear modify ankle biomechanics and foot loading patterns?

**DOI:** 10.1186/s13047-014-0040-y

**Published:** 2014-09-26

**Authors:** Carina Price, Vaidas Andrejevas, Andrew H Findlow, Philip Graham-Smith, Richard Jones

**Affiliations:** Centre for Health Sciences Research, University of Salford, Salford, M6 6PU UK; School of Health Sciences, University of Salford, Salford, M6 6PU UK

**Keywords:** Flip-flop, Gait, Electromyography, Loading rate

## Abstract

**Background:**

Flip-flops are an item of footwear, which are rubber and loosely secured across the dorsal fore-foot. These are popular in warm climates; however are widely criticised for being detrimental to foot health and potentially modifying walking gait. Contemporary alternatives exist including FitFlop, which has a wider strap positioned closer to the ankle and a thicker, ergonomic, multi-density midsole. Therefore the current study investigated gait modifications when wearing flip-flop style footwear compared to barefoot walking. Additionally walking in a flip-flop was compared to that FitFlop alternative.

**Methods:**

Testing was undertaken on 40 participants (20 male and 20 female, mean ± 1 SD age 35.2 ± 10.2 years, B.M.I 24.8 ± 4.7 kg.m^−2^). Kinematic, kinetic and electromyographic gait parameters were collected while participants walked through a 3D capture volume over a force plate with the lower limbs defined using retro-reflective markers. Ankle angle in swing, frontal plane motion in stance and force loading rates at initial contact were compared. Statistical analysis utilised ANOVA to compare differences between experimental conditions.

**Results:**

The flip-flop footwear conditions altered gait parameters when compared to barefoot. Maximum ankle dorsiflexion in swing was greater in the flip-flop (7.6 ± 2.6°, *p* = 0.004) and FitFlop (8.5 ± 3.4°, *p* < 0.001) than barefoot (6.7 ± 2.6°). Significantly higher tibialis anterior activation was measured in terminal swing in FitFlop (32.6%, *p* < 0.001) and flip-flop (31.2%, *p* < 0.001) compared to barefoot. A faster heel velocity toward the floor was evident in the FitFlop (−.326 ± .068 m.s^−1^, *p* < 0.001) and flip-flop (−.342 ± .074 m.s^−1^, *p* < 0.001) compared to barefoot (−.170 ± .065 m.s^−1^). The FitFlop reduced frontal plane ankle peak eversion during stance (−3.5 ± 2.2°) compared to walking in the flip-flop (−4.4 ± 1.9°, *p* = 0.008) and barefoot (−4.3 ± 2.1°, *p* = 0.032). The FitFlop more effectively attenuated impact compared to the flip-flop, reducing the maximal instantaneous loading rate by 19% (*p* < 0.001).

**Conclusions:**

Modifications to the sagittal plane ankle angle, frontal plane motion and characteristics of initial contact observed in barefoot walking occur in flip-flop footwear. The FitFlop may reduce risks traditionally associated with flip-flop footwear by reducing loading rate at heel strike and frontal plane motion at the ankle during stance.

## Background

Flip-flops are a popular summer shoe in the United Kingdom and commonly worn throughout the year in warmer climates such as America and Australasia [1,2]. This style of footwear is defined by having one strap across the dorsal fore-foot, which attaches to the footbed between the hallux and second toe to the thin, flexible sole. Despite the popularity of flip-flops, heel pain and other conditions such as overuse injuries of the tibialis anterior and toes have been associated with wearing flip-flop style footwear by podiatrists [3]. Flip-flops differ from standard walking footwear design due to a thin sole, no medial arch support, no protection for the toes, being loose fitting and having no pitch from heel to toe [4,5]. Despite these recognised potentially detrimental features, the popularity of flip-flops in warm climates continues. Nevertheless, limited scientific investigation into their influence on adult gait and associated pathologies has been published in peer-reviewed literature.

Descriptive research is evident quantifying gait in flip-flops, most extensively in children. Children wearing flip-flops display a trend towards a more dorsiflexed, everted and abducted midfoot during walking [6] and reduced hallux dorsiflexion prior to contact during walking and jogging [7]. Reduced eversion during midstance in adults has also been demonstrated when walking in flip-flops, compared to barefoot [8]. Video data has been used in an observational study of pedestrians, identifying a reduction in average walking speeds when walking in flip-flops [2], which was attributed to a shorter stride length compared with other footwear and confirmed in a laboratory environment [9]. In addition, an experimental study using 2D gait analysis concluded that there was an increase in ankle plantarflexion during swing, which the authors hypothesised could be due to contraction of the toe flexors to keep the flip-flop on the foot due to the lack of heel-strap or full upper [9]. Contrasting these findings, Chard *et al.* [6] identified greater ankle dorsiflexion prior to and at heel contact when walking in flip-flops compared to barefoot conditions.

Literature more specific to potential detrimental features of gait in flip-flops is uncommon and includes some methodological weaknesses. A plantar pressure study on females walking in flip-flops postulated that “gripping” with the toes occurs to hold this footwear on the foot, however the variable used to speculate this may not have been relevant [1]. Additionally, this study referred to potential benefits in terms of the shoe providing protection to the plantar surface of the foot, reducing plantar pressures compared to barefoot walking due to the material. In contrast, the impact attenuation that flip-flops provide at heel strike has been investigated by Zhang *et al.* [10], but no significant difference in loading rates between barefoot and flip-flops was evident. However, the parameter used in their investigation was not maximum instantaneous loading rate, which may have attributed to identified differences between conditions. Describing gait in flip-flops is useful, but more valuable is comparing the influence of these styles of footwear to a relevant and available replacement. This may enable the identification of a replacement item of footwear that fulfils the same purpose, however reduces or prevents the potentially detrimental gait modifications in the wearer.

The FitFlop was originally developed with a multi-density midsole to increase muscle activation in the lower limb by incorporating a soft mid-foot to induce instability [11]. This footwear encompasses a thick multi-density EVA sole with a wider and higher fitting flip-flop style upper. These features may reduce potentially detrimental gait modifications so making this footwear a more suitable alternative to a flip-flop. A recent paper identified that the FitFlop can reduce foot plantar pressures during walking when compared to a flip-flop [12]. However, gait motion analysis in this footwear compared to a flip-flop comparator is yet to be fully investigated to explore this alternative.

The current research study aims to compare barefoot walking to walking in flip-flop style footwear and walking in a FitFlop; and walking in FitFlop to walking in flip-flop to see if this contemporary footwear design offers a potential advantage in terms of gait modifications. Firstly, it is hypothesised that shod conditions will increase ankle dorsiflexion and tibialis anterior muscle activation during swing and at heel strike compared to barefoot to hold the footwear during swing. However, this increase in dorsiflexion is predicted to be less in FitFlop than flip-flop due to the size and position of the dorsal strap. Secondly, there is expected to be a reduction in the frontal plane motion of the foot in the FitFlop condition during stance compared to both barefoot and flip-flop due to the ergonomically profiled sole and the features of the dorsal strap. Thirdly, shod conditions are expected to attenuate force loading rates at heel strike compared to barefoot due to the inclusion of material under the calcaneus; with the FitFlop predicted to have a greater reduction in loading rate compared to the flip-flop due to its greater sole thickness.

## Methods

### Participants

Forty participants took part in the study, twenty female and twenty male (Table 1) recruited from the University staff and student population. All indicated they were asymptomatic i.e. had no diagnosed gait pathologies for at least three months, and gave written informed consent fulfilling the requirements of the University of Salford Research Ethics Panel.

**Table 1:** **Participant characteristics (mean ±** ***sd*** **)**

	**Overall**	**Male**	**Female**
Age (years)	35.2 ± 10.2	32.7 ± 9.0	37.7 ± 10.9
Mass (kg)	72.5 ± 15.2	81.6 ± 12.8	63.5 ± 11.8
Height (m)	1.71 ± 0.09	1.78 ± 0.07	1.64 ± 0.05
Body mass index (B.M.I. kg.m^−2^)	24.8 ± 4.7	26.1 ± 4.7	23.6 ± 4.4
U.K. shoe size	7 ± 2	9 ± 1	6 ± 1

### Footwear conditions

**Figure 1:**
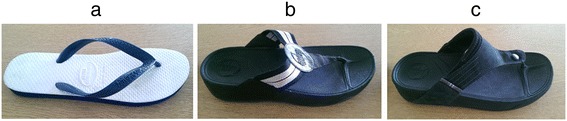
**Footwear conditions tested: Havaiana flip-flop (a), Female FitFlop, Walkstar I (b) and Male FitFlop, Dass (c).**

Testing utilised barefoot and two footwear conditions: flip-flop and FitFlop (Figure 1, Table 2). The FitFlop varied between genders as a single style does not span the size range of participants. These variations included the last shape (used in the manufacture), the dorsal strap material and the thicker sole (+4 mm) in the male version. The coverage and position of the dorsal strap on the foot however, were consistent between shoes. The male and female data were combined for comparison between conditions.

**Table 2:** **Footwear characteristics for an example male and female shoe size from each condition**

**Condition**	**Style**	**Midsole construction**	**Heel depth (mm)**	**Hardness (shore A)**	**Shoe mass (g)**
Flip-flop	Havaiana Brazil	EVA	Size UK 6: 16	33	Size UK 6: 140
			Size UK 9: 18		Size UK 9: 172
FitFlop	Female = FitFlop Walkstar I.	Multi-density EVA in heel, midfoot and toe. Rubber outsole.	Size UK 6: 33	Heel: 55	Size UK 6: 172
				Midfoot: 28	
				Toe 38	
	Male = Dass		Size UK 9: 37		Size UK 9: 270

Where EVA is ethylene vinyl acetate.

### Protocol

Participants undertook five walking trials for each condition, the order of which was randomised. Participants walked at their self-selected speed for each condition through the laboratory while kinematic, kinetic and Electromyography (EMG) data were recorded. Data from the right leg was used for analysis.

### Kinematics and kinetics

Three-dimensional motion data were collected using a 12 Camera Qualisys Opus system (Qualisys, Gothenburg, Sweden) sampling at 100 Hz. Spherical retro-reflective markers were positioned on the anatomical landmarks of the lower extremity to define the foot, leg, thigh, and pelvis. Rigid plates with reflective markers attached were fastened to the segments to enable the CAST technique to be utilised [13]. This technique uses a static trial recorded at outset, which enables the rigid plates to be defined relative to the anatomical landmarks for each segment. Joint angles were defined such that a static posture was zero and ankle dorsiflexion and inversion were positive. Kinetic data were collected simultaneously using two force platforms (Advanced Medical Technologies Inc., Newton, Massachusetts, USA) at 3000 Hz. These data were exported to Visual 3D (C-Motion Inc., Rockville, Maryland, USA) where kinematic and kinetic data were filtered using second-order Butterworth filters at 10 and 25 Hz respectively.

Visual 3D software was utilised to build a six degree-of-freedom model of the lower limbs. A pre-written pipeline was used to calculate kinematic and kinetic variables including joint angles and internal joint moments for the right limb normalised to body weight and gait cycle time where appropriate. Heel strike was defined at the point where the vertical GRF exceeded 10 N [14]. Peak values and magnitudes at heel strike and toe-off for relevant kinematic variables were identified for statistical analysis. GRF impulse and maximum instantaneous loading rate from heel strike to 65 ms were calculated to enable comparison of trials with and without heel strike transients consistently [15,16]. Walking speed was computed from kinematic data within Visual 3D.

### Electromyography

28 participants from the 40 were tested for muscle activation (mean ± 1 standard deviation, Male, N = 15, age = 30 ± 8 years, B.M.I = 25.9 ± 4.5 kg.m^−2^; Female, N = 13, age = 37.8 ± 12.4 years, B.M.I = 23.0 ± 4.7 kg.m^−2^) due to technical difficulties. EMG was recorded simultaneously at 3000 Hz using bipolar surface Ag/AgCl electrodes (Noraxon Inc, Scottsdale, Arizona, USA), with an electrode diameter of 10 mm and an inter-electrode spacing of 20 mm. Prior to electrode placement, hair was removed, skin exfoliated and cleaned. Electrodes were placed in accordance with the SENIAM recommendations on the tibialis anterior and peroneus longus [17]. The ground electrode was placed overlying the medial condyle of the tibia.

EMG analysis was undertaken in Visual 3D. Data was filtered to remove zero-offset (high-pass Butterworth 20 Hz) and a linear envelope was produced using a 10 Hz low-pass Butterworth filter. The linear envelope EMG was integrated (EMGLI) within the Gait Cycle events: initial contact (0–2%), loading response (0–10%), midstance (10–30%), terminal stance (30–50%), pre-swing (50–60%), initial swing (60–73%), mid-swing (73–87%) and terminal swing (87–100%) [18]. The gait cycle phases relating to the specific hypothesis above were compared only for the specific muscles.

### Statistical analysis

Variables were calculated on an individual participant basis from non-normalised data for statistical comparison averaged across trials then participants to produce an individual then group mean. Data is presented in text and figures as mean ± 1 standard deviation. Figures present ensemble average data normalised to gait cycle/stance time. SPSS (Version 17.0, SPSS Inc., Chicago, U.S.A.) software was utilised for statistical testing specific to study hypotheses, utilising between participants analysis of variance (ANOVA) to identify differences between conditions. Data that was not normally distributed (electromyography and joint moments) was square-root transformed, checked for normality, and treated as parametric. Holm adjustment for multiple comparisons was used and effect size (Cohen’s d, *d*) was reported for significant differences.

## Results

### Ankle angle swing

**Figure 2:**
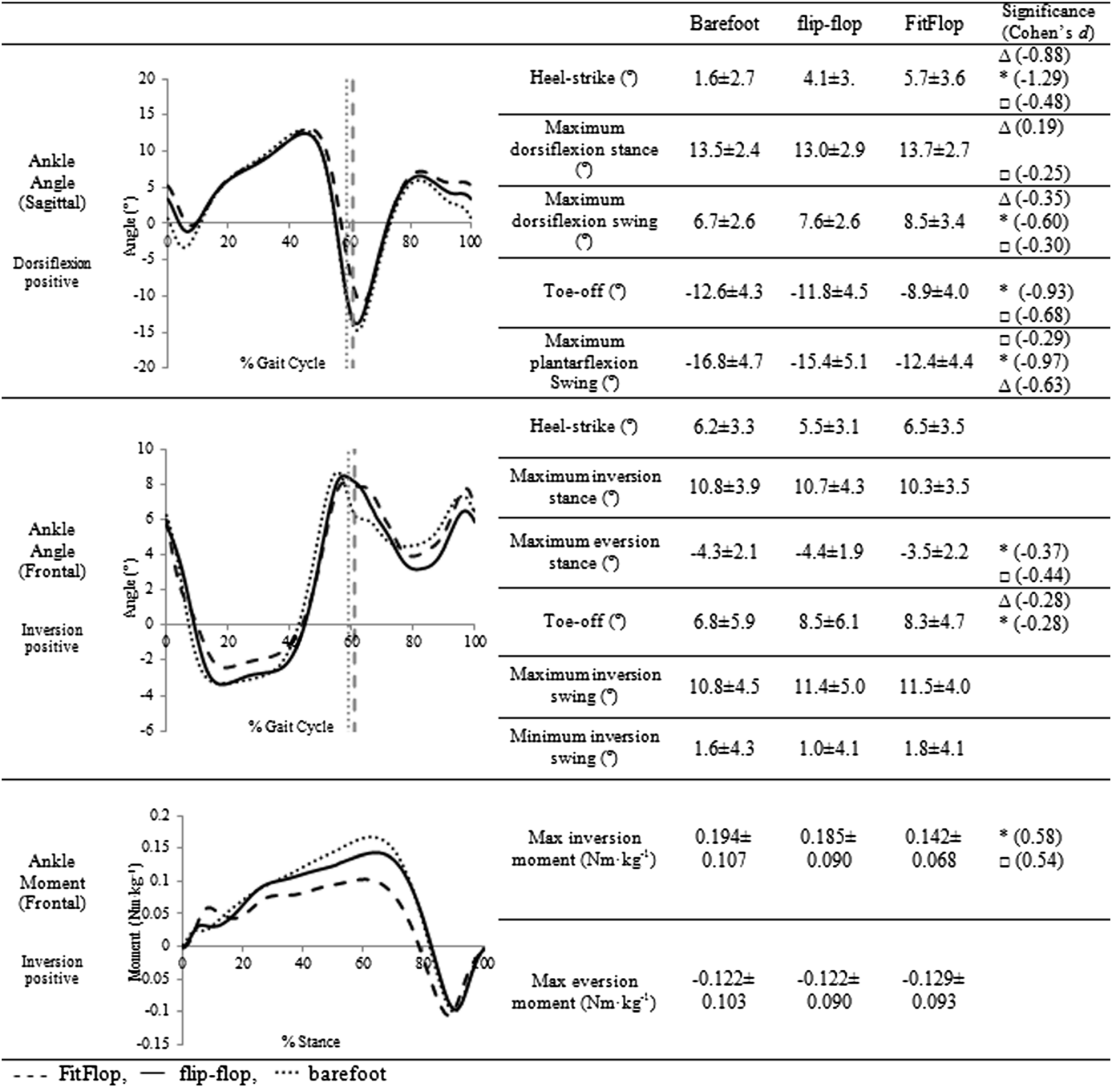
**Ensemble average ankle kinematics and kinetics.** Kinematics normalised to the gait cycle, kinetics normalised to stance time, where vertical lines denote toe-off. With values calculated prior to normalisation and presented as mean ± 1 standard deviation. Statistical significance denoted by: *barefoot significantly different to FitFlop, Δ barefoot significantly different to flip-flop. □ FitFlop significantly different to flip-flop.

**Figure 3:**
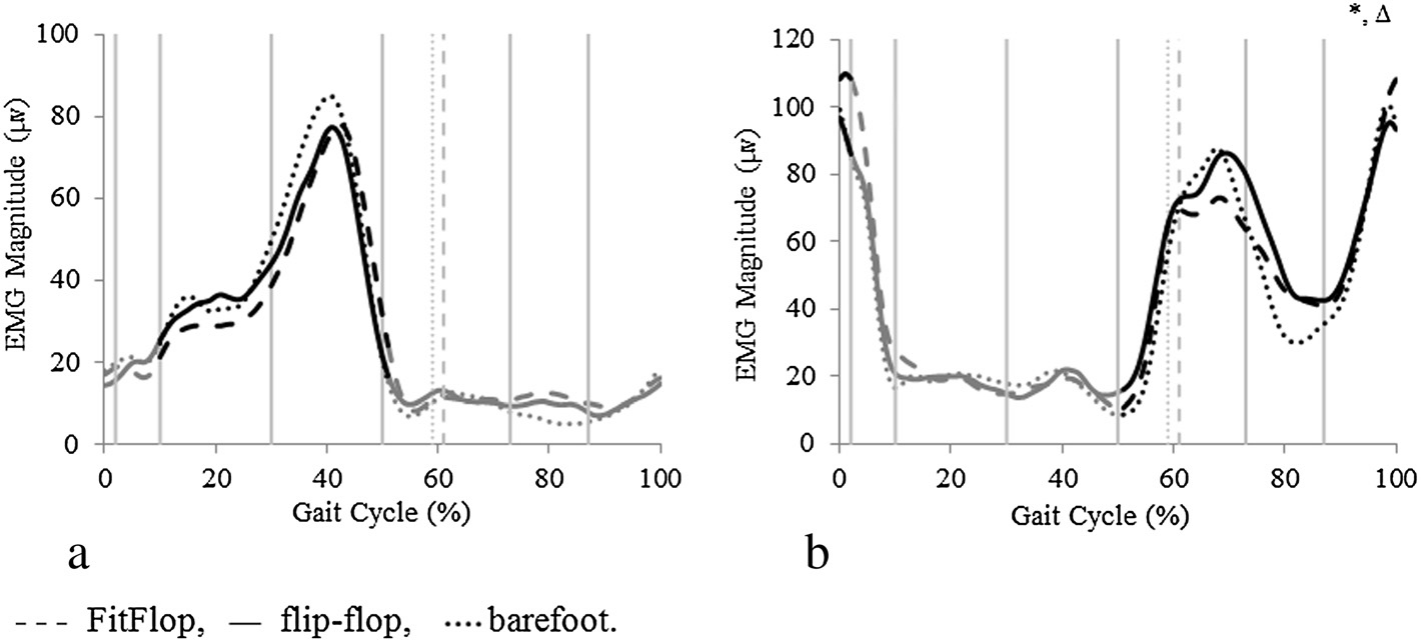
**Mean of all participant (N = 28) data for electromyography linear envelope (μv) normalised to the gait cycle for the a) peroneus longus b) tibialis anterior.** Vertical dashed lines denote toe-off and black highlighs regions that were compared statistically. Statistical significance denoted by: *barefoot significantly different to FitFlop, Δ barefoot significantly different to flip-flop. □FitFlop significantly different to flip-flop.

Ankle joint angles and muscle activation differed between conditions in the sagittal plane (Figures 2, 3). Peak dorsiflexion and plantarflexion angles in swing differed significantly between conditions with small to large effect sizes. Specifically, the flip-flop style footwear recorded greater (FitFlop 8.5 ± 3.4°, flip-flop 7.6 ± 2.6°, barefoot 6.7 ± 2.6°; Figure 2) maximum dorsiflexion values and reduced maximum plantarflexion angles (FitFlop −12.4 ± 4.4°, flip-flop −15.4 ± 5.1°, barefoot −16.8 ± 4.7°; Figure 2) compared to barefoot. Muscle activation measurement recorded significantly higher tibialis anterior activation in terminal swing in FitFlop (mean 32.6%, *p* < 0.001, *d* = −0.11) and flip-flop (31.2%, *p* < 0.001, *d* = 0.27) compared to barefoot (Figure 3).

### Frontal plane ankle in stance

In the frontal plane the FitFlop reduced the range of motion compared to the other conditions by approximately 10% (Figure 2). Maximum eversion was significantly lower in FitFlop (−3.5 ± 2.2°) compared to in the flip-flop (−4.4 ± 1.9°, *p* = 0.008, *d* = −0.44) and barefoot (−4.3 ± 2.1°, *p* = 0.032, *d* = −0.37) conditions. Alongside alterations in joint angle, significantly lower inversion moment was recorded during late stance in FitFlop (0.142 ± 0.068 Nm.kg^−1^) than flip-flop (0.185 ± 0.090 Nm.kg^−1^, *p* < 0.001, *d* = 1.04) and barefoot (0.194 ± 0.107 Nm.kg^−1^, *p* < 0.001, *d* = 1.36) (Figure 2). Peroneus longus muscle activation did not differ between conditions during stance (Figure 3).

### Loading rate

The nature of the impact with floor differed between the three conditions with significant differences evident in the GRF variables as well as vertical heel velocities toward the floor. The maximum loading rate significantly differed between conditions with both the flip-flop (26.7 ± 5.6 BW · s^−1^) and FitFlop (21.7 ± 5.4 BW · s^−1^) providing significant reductions compared to Barefoot (41.4 ± 22.9 BW · s^−1^, *p* < 0.001, *d* > 0.91). Furthermore, the FitFlop condition reduced loading rate by 19% compared to flip-flop (*p* < 0.001, *d* = 0.88). The impulse of the vertical GRF from heel strike to 65 ms was significantly lower in FitFlop (.029 ± .006 BW · s, *p* < 0.001, *d* = 1.09) and flip-flop (.034 ± .005 BW · s, *p* = 0.032, *d* = 0.20) than barefoot (.035 ± .005 BW · s). These were despite the walking speed being significantly higher in the FitFlop (1.32 ± .10 m.s^−1^) compared to the flip-flop (1.29 ± .11 m.s^−1^) condition. The vertical heel velocity at heel strike was significantly faster toward the ground in the flip-flop style footwear (flip-flop: −.342 ± .074 m · s^−1^, *p* < 0.001, *d* = 2.5; FitFlop -.326 ± .068 m · s^−1^, *p* < 0.001, *d* = 2.3) than the barefoot condition (−.170 ± .065 m · s^−1^).

## Discussion

This study has undertaken an assessment of the biomechanics of gait when walking in flip-flop style footwear and compared it to barefoot walking. The standard flip-flop was also assessed in comparison to a contemporary version of this style of footwear; the FitFlop. The research has highlighted statistically significant differences in ankle angle in swing, frontal plane motion and loading rate of the vertical ground reaction force, while walking in a standard flip-flop and FitFlop compared to barefoot walking. Contrasting gait patterns are evident in the FitFlop, which may pose advantages to the wearer of flip-flop style footwear. Walking speeds in all conditions in the present study were comparable to the speed that people walk in flip-flops in their daily lives (1.31 m.s^−1^) [2]. This suggests that results are generalizable to adults walking in flip-flop and FitFlop footwear in a real-world environment.

### Ankle angle swing

As hypothesised, the flip-flop style conditions demonstrated moderations to sagittal plane motion at the ankle joint motion at heel strike, toe off and during swing compared to barefoot. This trend toward dorsiflexion, or reduced plantarflexion, in shod conditions is consistent with previous literature [6], particularly during swing, and is potentially a mechanism to keep the shoes on the foot. The dorsal strap for both footwear conditions only covers the front of the foot and thus, gait may be adapted to hold the shoe on the foot [9]. In contrast to the current study, Shroyer *et al.* identified increased plantarflexion in swing when participants wore flip-flops compared to trainers [9]. The authors attributed their finding to contraction of the toe flexors to hold the flip-flop, creating a plantar-flexor moment at the ankle, however no electromyography data was collected. Consistent with this prediction, a recent plantar pressure analysis identified pressure under the hallux in swing in both flip-flops and FitFlops, which was attributed to gripping [12]. The FitFlop demonstrated reductions in magnitude and duration of gripping compared with flip-flop [12], potentially reducing any resultant plantar-flexor moment at the ankle and therefore enabling greater dorsiflexion compared to the flip-flop. Inferences from the current data and literature imply that ankle dorsiflexion and toe flexion may combine to hold toe-post footwear on the foot during swing, however toe motion must be quantified to confirm this. Contradicting the original hypothesis, the FitFlop increased dorsiflexion in swing and tibialis anterior activation compared to flip-flop, as opposed to reducing this mechanism. This may be due to the aforementioned reduced toe-flexor moment, or the increased mass and thicker sole of this shoe requiring greater ground clearance than the flip-flop condition. Results from the present study demonstrate significantly higher tibialis anterior activation in shod conditions than barefoot in terminal swing, consistent with the increase in dorsiflexion in swing and reports from other authors [10].

### Frontal plane ankle in stance

As anticipated, in the frontal plane the FitFlop reduced the joint excursion compared to other conditions by approximately 10%, in particular eversion was reduced during stance (Figure 1). The flip-flop condition showed consistent patterns to barefoot as would be expected with a flat, flexible sole and a thin, loose fitting upper (Figure 1), foot motion is unchanged during stance [19]. Previous studies are inconsistent reporting increased midfoot eversion [6], no significant differences in frontal plane range-of-motion [10] and reduced eversion in midstance in a flip-flop compared to barefoot [8]. The FitFlop thicker upper and soft profiled footbed therefore appear to interact to control frontal plane motion of the ankle and tarsal joints when compared to a standard flip-flop design. This may be potentially beneficial to wearers and may in-part, explain positive testimonials from consumers as excessive frontal plane motion of the ankle has been repeatedly linked to overuse injuries [20-22]. A significantly reduced inversion moment was recorded in FitFlop throughout stance, with a significantly lower peak moment in terminal stance than both flip-flop and barefoot. This reduction may be attributed to the less everted foot position reducing the distance between the GRF and the ankle joint centre [20]. Increased ankle external eversion moments have been linked to increased injury potential in running [20].

The flip-flop peak inversion moment in the current study was equivalent to that in barefoot. This contrasts previous research, which has identified increased maximum external inversion moment in a flip-flop compared to other footwear conditions and barefoot [23]. This may have been due to pathology related motion present in one of the previous studies knee osteoarthritic population [23]. Similarly, another study reports reduced peak ankle inversion moment in late stance in flip-flops compared to barefoot in a male population [10]. This may be a function of gender, familiarity with the footwear or differences between the specific styles utilised in the study. Gender differences have previously been identified in sagittal ankle angle walking in flip-flops [9] and future work should consider both gender differences and footwear style familiarity differences to clarify any gait modifications in flip-flop style footwear in specific groups. This is a limitation of the current research, as any evident differences between genders could not be isolated to gender alone as opposed to footwear differences in the styles; interactions were not compared.

### Loading rate

The flip-flop was expected to reduce loading rate at ground impact compared to barefoot and the FitFlop was expected to further reduce this loading, both of which were confirmed. Analysis of the GRF was designed to allow comparison of heel strike loading features when not all trials included a transient feature. Loading rates quantified in the present study were consistent with previous literature for the shod values, barefoot values were lower than the 117.8 ± 27.5 BW.s^−1^ reported in previous literature, however this literature utilised a fixed walking speed of 1.5 m.s^−1^, faster than the current study [15,16].

Velocity of the heel toward the floor was twice as fast in the flip-flop style conditions compared to the barefoot, consistent with previous findings in flip-flops and sandals [24,25]. Explanation of increased heel velocity in this shoe style may be a result of kinematic changes due to the upper, proprioceptive due to the shoe leaving the foot at the heel or due to protective kinematic adaptations in barefoot gait to reduce impact energy, as evident in running [25,26]. Despite the higher heel velocity and therefore higher impact energy in both flip-flop style conditions, the force loading rate was lower than in barefoot. This contrasts previous research which reports no difference in loading rate between barefoot and flip-flop conditions, but calculated loading rate to loading peak of GRF as opposed to the maximum instantaneous value [10]. This may have masked differences at heel-contact due to the loading peak of the GRF largely being a function of body mass, kinematics and walking velocity as opposed to features of the footwear or heel velocity. The flip-flop would be expected to attenuate shock at initial contact compared to barefoot as a layer of EVA is placed under the foot. Viscoelastic material absorbs energy and therefore can attenuate the impact of the foot with the floor [27,28], whereas the barefoot condition only has the internal structures of the foot as attenuating materials. Similarly, a plantar pressure study has suggested that results show the flip-flop protecting the body at heel strike compared to barefoot [1]. The FitFlop absorbed greater shock at heel strike, evident by 19% and 15% reductions in loading rate and impulse compared to flip-flop, with strong effect. This is likely due to the softer and thicker construction of EVA in the heel section of the FitFlop compared to flip-flop (Table 2); thickness being the most important factor when considering shock absorption properties of viscoelastic materials [27]. Reduced loading rate of the ground reaction force likely reduces the potential for skeletal injury during walking [28,29].

## Conclusions

The current study identified increased ankle dorsiflexor activity in flip-flop style footwear compared to barefoot, coupled with increased dorsiflexion in swing, assumed to be a mechanism to hold the shoe on the foot. The FitFlop limited foot motion in the frontal plane and significantly reduced loading at impact, compared to flip-flop and barefoot. However, it is not clear whether the reductions in these parameters are enough to reduce any potential injury or overuse injuries associated with flip-flop footwear and further, longitudinal, research would be needed to clarify this relationship.
